# Minimum Normalized Cycling Cadence to Increase Post-Cycling Gait Velocity

**DOI:** 10.3390/jfmk9040235

**Published:** 2024-11-13

**Authors:** Nitu Lama, Christopher J. Keating, Paul T. Donahue, Nuno Oliveira, Tanner A. Thorsen

**Affiliations:** 1 School of Kinesiology and Nutrition, The University of Southern Mississippi, Hattiesburg, MS 39406, USA; nitu.lama@usm.edu (N.L.);; 2 Facultad de Deportes, Universidad Católica San Antonio de Murcia, 30107 Murcia, Spain

**Keywords:** gait velocity, gait analysis, cycling, kinematics, kinetics

## Abstract

Background: Previous research has shown that increasing cycling cadence can result in improved post-cycling gait velocity. However, the specific threshold of cycling cadence required to bring about clinically meaningful changes in gait velocity remains unknown. This study aimed to determine the minimum increment in cycling cadence that would lead to a significant improvement in post-cycling gait velocity. Methods: A total of 42 young adults participated in our study and were randomly assigned to one of three groups: TEN, TWENTY, and THIRTY. Each group was assigned to cycle at a cadence at the corresponding percentage higher than the participant’s self-selected gait cadence. Each participant engaged in a 15-min cycling session at their respective assigned cycling cadence. Before and after cycling, participants completed a 10-meter walk test while spatiotemporal parameters of gait, ground reaction forces, lower extremity kinematics, and kinetics were recorded. Results: One-way ANOVA revealed no statistically significant changes in spatiotemporal, ground reaction force, kinematics, and kinetics variables pre- and post-cycling. However, there were both statistically significant (F(2,41) = 3.794, *p* = 0.031, η^2^ = 0.604) and clinically meaningful changes (0.07 m/s) in post-cycling gait velocity in the THIRTY group only. Conclusions: This suggests that a cycling cadence of 30% or higher is the minimum requirement to produce a clinically significant improvement in gait velocity.

## 1. Introduction

Gait velocity, also known as walking speed, is recognized as a functional measure and is often referred to as the “sixth vital sign”. Like heart rate or blood pressure, it responds to various factors such as disease, cognition, and training status [1,2]. This sensitivity to changes in physiological status highlights the importance of gait velocity as a valuable indicator in assessing an individual’s functional abilities and overall health. Gait velocity, for example, has been recognized as a valuable predictor of fall risk in older adults [3], and improving gait velocity and walking ability may be an effective technique for reducing fall incidence. Improving gait ability has also been shown to be important in various other populations, including aging [4], multiple sclerosis [5], Parkinson’s disease [6], and spinal cord injury [7]. Previous works suggest that clinically meaningful improvement in gait velocity can be considered with increases between 0.05 to 0.1 m/s [8,9].

Research studies have shown that the tasks of walking and stationary cycling are inherently similar, in that both activities elicit comparable neuromuscular facilitation in the lower extremities, as evidenced by brain activation studies during walking and pedaling [10,11]. For example, in both tasks, coordinated activation of the plantar flexors, knee extensors, and hip extensors are required for the forward propulsion of the center of mass. Cycling also provides additional benefit to the lower extremities as it requires a wider range of motion of the lower extremity joints than walking, which may be viewed as a benefit in maintaining or improving flexibility and strength in joints, contributing to more efficient walking mechanics [12].

Stationary cycle ergometers offer a low-impact, cost-effective, and safe exercise option for improving health parameters. This low-impact nature of stationary cycling has proven to be especially beneficial in improving various gait parameters for individuals with Parkinson’s disease, multiple sclerosis, or those undergoing stroke rehabilitation [13,14,15]. Furthermore, the versatility of stationary cycle ergometers allows for indoor and outdoor use, making it a year-round activity option. Recent research studies have demonstrated that cycling can enhance gait cadence and gait velocity acutely post-cycling in older individuals with Parkinson’s disease [16,17]. Cycling also allows for graded increases in work rate to be applied to the lower extremities and reduces the risk of falling while performing the exercise. This makes cycling a particularly appealing exercise modality for individuals of whom walking may not be a safe task, or other resistance training modalities may not be reasonable.

Cycling cadence is measured as the number of revolutions of one pedal per minute (RPM), whereas gait cadence is measured as the number of steps (both right and left legs) taken per minute (steps per minute, SPM). Dividing SPM in half approximates comparable movement frequency of each leg during walking and running and allows researchers a comparable metric to evaluate walking and running with cycling from a movement-frequency-synchronized perspective. It has been shown that cycling at a higher cadence than one’s self-selected gait cadence leads to an increase in gait velocity [1]. In a recent study by Keating and colleagues [1], increased cycling cadence was shown to lead to an average increase in gait velocity of 0.1 m/s in younger adults. However, it is important to note that cycling at a higher work rate did not have the same effect on gait velocity, suggesting that an increase in cycling cadence is necessary to achieve an increase in gait velocity. In this investigation, participants cycled at a cadence of 75 RPM, analogous to a 36% increase in gait cadence from the typical 110 SPM, and demonstrated an 8.4% increase in post-cycling gait velocity [1]. The increase in gait velocity was accomplished by an increase in gait cadence, not stride length, suggesting participants walked faster because they took more, but not longer, steps [1]. These findings were consistent with a similar study conducted by Tsushima et al. [16] who reported an 8% increase in gait velocity when cycling cadence was increased by 18% above the self-selected cycling cadence [16].

The results of these studies suggest a potential positive impact of cycling on subsequent walking performance in terms of velocity; however, a number of questions regarding the mechanism underlying the observed increase in post-cycling gait velocity remain. For example, both Keating et al. [1] and Tsushima et al. [16] had participants complete bouts of cycling exercise at cadences that were 36% and 18% greater than their walking cadence, respectively, and both demonstrated clinically meaningful increases in post-cycling gait velocity [1,16]. With an eye looking forward to future implementation of cycling as an intervention for the improvement of gait velocity, determining the minimum increment in cycling cadence over self-selected gait cadence will improve our understanding of the dose and response relationship between high-cadence cycling and gait velocity. In addition, both Keating et al. [1] and Tsushima et al. [16] asked participants to complete bouts of cycling at absolute cadences (e.g., 75 RPM) without direct consideration of individual variation in pre-cycling gait cadence This study aimed to address gaps in the previous research, which did not account for individual differences in pre-cycling gait cadence, nor investigate any between-sex differences observed following high-cadence cycling. By normalizing cycling cadence to each participant’s self-selected gait cadence, we sought to provide more accurate insights into the individualized effects of high-cadence cycling on gait velocity.

Thus, the purpose of this study was to determine the minimum increase in cycling cadence—as normalized to individual self-selected gait cadence—that would elicit a statistically and clinically meaningful acute increase in gait velocity. Additionally, we attempted to examine changes in lower extremity biomechanics associated with changes in gait parameters. Based on our previous laboratory work and the previously reported literature, we suggested the following three hypotheses would be supported: (1) the minimum normalized increment in cycling cadence required to significantly and meaningfully increase gait velocity would be lower than 18% above self-selected SPM, (2) incrementally increasing cycling cadence above self-selected SPM would result in a non-linear increase in post-cycling gait velocity and spatiotemporal parameters of gait across all experimental conditions, and (3) there would be no difference in post-cycling gait velocity between males and females.

## 2. Materials and Methods

### 2.1. Participants

A random sample of 42 recreationally active young adults from the local community was recruited through word of mouth to take part in a one-time laboratory visit. Prior to participation, all individuals provided written informed consent, which was approved by the University Institutional Review Board.

Participants were included if they self-reported as being recreationally active for the last 3 months in accordance with the ACSM (American College of Sports Medicine) Guidelines for Physical Activity. This includes either 150 min of moderate-intensity aerobic activity per week, or muscle-strengthening activities on 2 or more days a week that consist of working all major muscle groups [18].

Potential participants were excluded from the study if they had sustained any lower extremity injuries within the last 6 months, received any major lower extremity surgery, presented a history of cardiovascular problems, or presented with a body mass index (BMI) greater than 40 kg/m^2^. Additional exclusion criteria included presentation of any neurological disorders or the use of assisted walking devices, such as prosthetic limbs or prophylactic braces. Finally, participants were screened using the PAR-Q (Physical Activity Readiness Questionnaire) to ensure safety during exercise.

### 2.2. Experimental Procedures

Upon arrival at the laboratory, participants’ height was measured with a stadiometer. Participants were prepared to complete all walking and cycling trials within a motion capture volume by having 18 retroreflective anatomic markers and 8 segmental tracking markers placed onto anatomical locations of interest. Bilateral placement of anatomical markers included the iliac crest, greater trochanter, medial and lateral femoral epicondyles, medial and lateral malleoli, distal end of the second toe, and the first and fifth metatarsal heads. Rigid thermoplastic shells were used to place segmental tracking marker clusters on the trunk, pelvis, thighs, shanks, and heels. Prior to any data collection, a static standing trial was recorded while the participant stood motionless in the motion capture volume on two force platforms, allowing for individual mass to be recorded. This static standing trial was then used as a calibration trial to obtain joint center definitions and segmental measurements. Following the static standing trial, the 18 anatomic markers were removed, leaving the segmental tracking clusters affixed to each segment [1,19].

Participants were then instructed how to complete a ten-meter walk test (10MWT) at a self-selected gait cadence. This was accomplished along a 30 m level walkway in which timing gates recorded the middle 6 m of the test [20]. The middle 6 m of the 10MWT was centered within the motion capture volume such that marker trajectories and ground reaction forces could be simultaneously recorded, for a minimum of three consecutive steps. Participants were given sufficient opportunity to practice the task, and when prepared, completed three consecutive trials of the 10MWT while kinematic marker trajectories and GRF were recorded. The duration for each participant to walk the middle 6 m of the 10MWT was obtained using two timing gates and was used to calculate 10MWT gait velocity.

Following these three 10MWT, the researchers processed the kinematic marker trajectories, and spatiotemporal parameters of gait were calculated [19]. Self-selected walking cadence was determined using the mean cadence recorded during three 10MWT trials. Then, a single 15 min bout of cycling was performed. Participants were randomly assigned to one of three cycling cadence groups. The first group had seven males and seven females and were instructed to cycle at a 10% increase in cycling cadence relative to the individual’s selected walking cadence (TEN). The second group had eight males and six females and were instructed to cycle at a 20% increase to the individual’s selected walking cadence (TWENTY). The third group had six males and eight females and were instructed to cycle at a 30% increase to the individual’s selected walking cadence (THIRTY). Immediately after the 15 min cycling bout, post-cycling gait parameters were recorded as participants performed three additional post-cycling 10MWT in a similar manner to the pre-cycling 10MWT.

### 2.3. Instrumentation

A six-camera Qualisys motion capture system was used to record three-dimensional marker coordinate data of the lower extremity (240 Hz, Qualisys, Gotenburg, Sweden). Concurrently, ground reaction force (GRF) signals were sampled at a frequency of 1200 Hz using six in-ground force plates (American Mechanical Technology Inc., Watertown, MA, USA), allowing for the GRF of three consecutive steps to be recorded.

Participants performed cycling on a mechanically braked cycle ergometer (828e, Monark, Vansbro, Sweden). Saddle height was adjusted to achieve a knee flexion angle between 25° and 30°, measured with a handheld goniometer when the pedal was at the bottom dead center position [21,22]. The saddle’s fore/aft position was adjusted to align the participant’s knee with the pedal spindle when the crank was in the forward horizontal (90°) position. The handlebar position was set to ensure a 90° angle between the trunk and thigh, also measured with a handheld goniometer when the crank was at 90°. Gait velocity was measured using a two-gate photocell timing system (Blue, Dashr, Lincoln, NE, USA) over a 6 m walkway [20].

### 2.4. Data Processing

Raw three-dimensional marker trajectories were first inspected and any gaps in marker trajectory were filled using relational interpolation of rigid body definitions of the segmental tracking clusters in Qualisys Track Manager. From there, processed marker trajectory and analog force signals were exported for analysis in the Visual 3D biomechanical analysis suite (Version 6.0, C-Motion; Germantown, MD, USA). A zero-lag fourth-order Butterworth low-pass filter was used to filter kinematic and GRF data at 6 Hz [23]. These data were then applied to a standard lower extremity musculoskeletal model [24] from which the spatiotemporal parameters of gait, ground reaction forces, lower extremity joint and segmental kinematics, and joint kinetics were determined. The spatiotemporal parameters obtained in this study were cadence, double-limb support time, stride length, and stride width. The stance phase of each step was specifically defined between heel strike (the initial occurrence when the vertical GRF surpassed a predefined threshold of 10 N on the force platform) and toe-off (the first instance when the vertical GRF dropped below a predetermined threshold of 10 N on the force platform) [1]. To define angular kinematic and kinetic variable conventions, angular computations were completed using a Cardan rotational sequence (X-Y-Z) based on the right-hand rule [25]. Positive rotations included ankle dorsiflexion and inversion, knee extension and adduction, and hip flexion and adduction. Internal joint moments were computed and expressed in the joint coordinate system [25]. GRF was normalized to body weight and joint moments were normalized to body mass. Peak joint angles were measured during the stance phase of the right leg. Peak GRF and joint moments were measured during the stance phase of one right foot. As participants performed all 10MWT within the motion capture volume, spatiotemporal characteristics were computed using data from both limbs.

The coefficient of variation (CV) was calculated as a method to ascertain meaningful change in post-cycling gait velocity at the level of each individual participant. We computed CV as the quotient between the mean and standard deviation of the three pre-cycling 10MWT times; thus, the CV indicated the variability in self-selected gait velocity for each individual [26]. Using the difference between the CV and pre-cycling mean of the 10MWT times as a lower bound, and the sum of the CV and pre-cycling mean of the 10MWT times as an upper bound, we established a window of expected variability for each participant’s pre-cycling self-selected gait velocity. Individual post-cycling 10MWT times were evaluated against this window of expected variability to determine if their post-cycling gait velocity fell below the lower bound of the window of expected variability, thus providing a participant-specific indication of meaningful increase in gait velocity.

### 2.5. Statistical Analysis

Our primary variables of interest were the change in gait velocity (the difference between POST and PRE cycling gait velocity) and the CV for each participant following the bout of cycling. Secondary variables included PRE-to-POST change in cadence, stride length, and stride width. Our tertiary explanatory variables were PRE-to-POST change in peak stance phase joint angles, peak stance phase joint moments, and peak stance phase GRF in all three planes.

Simple linear regression techniques were used to determine the relationship between increased cycling cadence and increased post-cycling gait velocity. For comparison, data from the FAST group of Keating et al. [1] was employed [1]. A one-way ANOVA was used to compare anthropometric, primary, secondary, and tertiary variables across the three different groups. Post hoc pairwise t-tests with a Bonferroni correction were performed to determine the location of statistical significance in the event of a significant main effect. A Mann–Whitney U test was used to compare the gait velocities (pre-cycling, post-cycling, and absolute change in gait velocity) between males and females to determine between-sex differences in gait velocity, as evidence from previous studies has shown sex-specific variations in kinematics and kinetics aspect of gait [27,28]. Statistical significance was established at α = 0.05. To determine the effect size of the repeated measures ANOVA tests, eta squared (η^2^) was computed and interpreted using effect sizes of 0.0099, 0.0588, and 0.1379 as small, medium, and large, respectively [29,30]. SPSS software (version 27, SPSS, Chicago, IL, USA) was used to perform all statistical analyses.

## 3. Results

A comparison of participant demographics between groups is presented in Table 1. There were no significant differences between participant demographics for any variable (Table 1).

Gait velocity increased in the TEN (Pre: 1.25 m/s, Post: 1.28 m/s), TWENTY (Pre: 1.19 m/s, Post: 1.23 m/s), and THIRTY (Pre: 1.13 m/s, Post: 1.20 m/s) groups; however, only the THIRTY group demonstrated a statistically significant and clinically relevant increase compared to the TEN and TWENTY groups (0.07 m/s, F(2,41) = 3.794, *p* = 0.031, Table 2).

**Figure 1:**
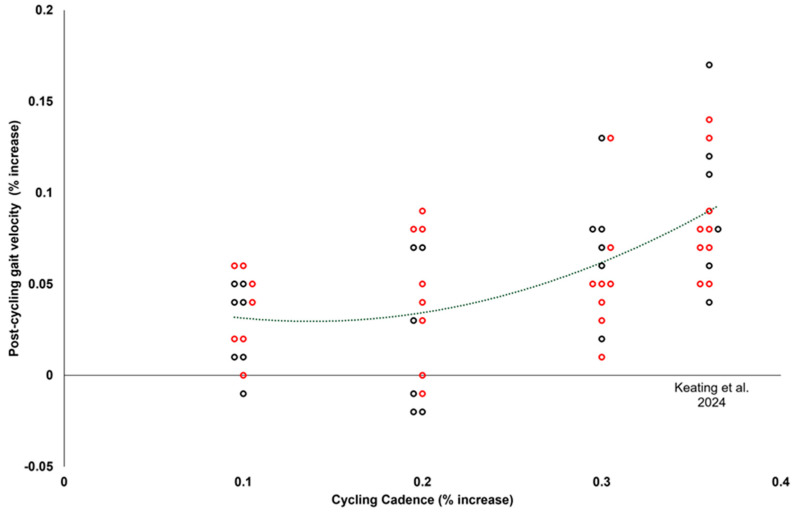
Relationship between cycling cadence (% increase from self-selected gait cadence) and post-cycling gait velocity (% change from pre-cycling gait velocity) of males (red) and females (black), defined by the equation y = 1.1778x^2^ − 0.317x + 0.0512, r^2^ = 0.336, *p* < 0.001. Individual data from the FAST group of Keating et al. [1] is included.

The relationship between increased cycling cadence and gait velocity was best described by a second-order polynomial equation (Figure 1). Using this model, increased cycling cadence accounted for 33.6% of the variance in post-cycling gait velocity (*p* < 0.001). The Mann–Whitney U test did not reveal any statistically significant variations in the gait velocities between males and females ((*p* > 0.005), Table 2, Figure 1).

**Figure 2:**
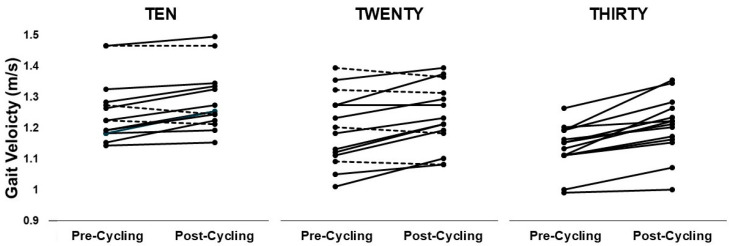
Participants who exceeded (smooth line) or did not exceed (dotted line) coefficient of variation (CV) window in each group.

With regards to CV, 21% of TEN and 36% of TWENTY did not walk with a post-cycling gait velocity that fell outside of their window of expected variability (Figure 2). However, in the THIRTY group, 100% of the participants walked with a post-cycling gait velocity that was outside the window of expected variability (Figure 2).

The increase in gait velocity observed in the THIRTY group was accompanied by increased cadence (F (2,41) = 2.626, *p* = 0.035, Table 2), as well as decreased double limb support time (F (2,41) = 4.228, *p* = 0.022, Table 3), suggesting that participants were taking more, but not necessarily spatially longer steps following cycling exercise.

No significant changes were observed for any GRF, but we did observe a significant reduction in peak vertical loading response (LR) GRF for the TWENTY group (F (2,41) = 6.460, *p* = 0.004, Table 4). Post-hoc analysis indicated that peak LR GRF for the TWENTY group was reduced compared to TEN group (*p* = 0.004) and the THIRTY group (*p* = 0.047).

We did not observe any statistically significant kinematic changes at the ankle, knee, or hip (Table 5). We did, however, observe a statistically significant difference in peak knee extension moment (F (2,41) = 5.317, *p* = 0.009, Table 6). Post-hoc analysis suggests a decrease in peak knee extension moment for the TWENTY group when compared to the TEN (*p* = 0.010) and the THIRTY (*p* = 0.045) groups.

## 4. Discussion

The purpose of this study was to determine the minimum increase in cycling cadence—as normalized to individual self-selected gait cadence—that would elicit a statistically and clinically meaningful increase in gait velocity. Additionally, we attempted to examine changes in lower extremity biomechanics associated with changes in gait parameters. Our first hypothesis, that the minimum normalized increment in cycling cadence required to significantly and meaningfully increase gait velocity would be lower than 18% above self-selected SPM, was unsupported. The THIRTY group showed both a statistically significant and clinically relevant increase in gait velocity (from 1.13 m/s to 1.20 m/s) compared to the TEN and TWENTY groups. These findings align with observations made by Keating et al. [1], who reported similar improvements in post-cycling gait velocity within their “Fast” group, which cycled at a cadence 36% above the typical gait cadence [1]. The relationship between increased cycling cadence and gait velocity was best described by a second-order polynomial equation, accounting for 33.6% of the variance in post-cycling gait velocity, with a notable shift observed from the THIRTY group onwards. In addition, 100% of the THIRTY group walked with velocities faster than their pre-cycling gait velocity CV, compared to 21% of TEN and 36% of TWENTY. These findings collectively suggest that a 30% increase in cycling cadence is the threshold necessary to achieve statistically significant and clinically meaningful improvements in post-cycling gait velocity.

Our second hypothesis that a gradual increase in cycling cadence would result in a nonlinear improvement in post-cycling gait velocity was supported by our findings, which revealed that a second-order polynomial function most accurately represented the observed increment in gait velocity following cycling. These findings are consistent with previous studies [1,16], which have also reported improvements in gait velocity following high-cadence cycling. This aspect of cycling intervention is particularly noteworthy, as even brief, low-impact bouts of exercise have been shown to produce significant improvements in gait velocity. This finding contrasts with earlier studies, which typically employed longer-duration exercise sessions with higher work rates to elicit meaningful changes in gait parameters in various populations [13,14,15]. The ability to achieve similar outcomes with shorter, less intense interventions highlights the potential for more accessible and feasible rehabilitation strategies, particularly for individuals with physical limitations or reduced exercise tolerance.

The findings showed that increased gait velocity in the THIRTY group was accompanied by increased cadence and decreased double limb support time, indicating participants took more, but not longer, steps following the bout of high-cadence cycling. Keating et al. [1] also reported an increase in cadence [1]. In addition to this finding, our study observed a decrease in double limb support time, indicating a further enhancement in gait dynamics following the cycling intervention. This suggests that not only does cadence increase with cycling, but there is also a reduction in the duration during which both feet are in contact with the ground, contributing to increased velocity. The reduction in double limb support time, the phase when both feet are in contact with the ground, implies that participants were spending less time in a stable position, and this reflects a more dynamic and efficient gait pattern. This change likely resulted in increased gait velocity by reducing the time spent in transitional phases between steps.

We saw no significant changes in GRFs, except a reduction in peak vertical LR GRF for the TWENTY group. We also observed no significant kinematic changes at the ankle, knee, or hip, but there was a significant decrease in peak knee extension moment for the TWENTY group compared to the other groups. The small incremental increase in cycling cadence percentage between all groups (e.g., only 10% increase in cycling cadence between groups) may have overlapped the magnitudes of the variables between the groups, potentially masking any variations. We anticipate that this overlap in cadence adjustments might have limited the ability to detect more pronounced changes in both kinetic and kinematics variables, suggesting that larger increments in cycling cadence could be necessary to produce more detectable biomechanical changes in gait.

Our final hypothesis, that there would be no difference in post-cycling gait velocity between males and females, was supported by our findings. Previous work has suggested sex-based differences in the kinematic and kinetic characteristics of the lower extremities, particularly in the sagittal plane [27,28]. Such differences are thought to manifest in various phases of movement, especially during activities like walking, where joint angles, ranges of motion, and force generation may differ between males and females. In light of these findings, we aimed to investigate whether similar sex-specific differences would emerge in gait patterns, at least in terms of gait velocity, when participants walked at a self-selected cadence under both pre-cycling and post-cycling conditions. Contrary to the previously suggested sex differences, we did not observe any significant sex-specific variations in these parameters between pre- and post-cycling conditions, as the Mann–Whitney U test revealed no significant sex-specific variations in term of gait velocities. However, our results were consistent with those of Keating et al. [1], who also reported no significant changes in post-cycling gait velocity [1].

Nevertheless, our finding that a 30% increase in cycling cadence is the minimum threshold required to achieve significant improvements in gait velocity is of substantial importance for populations where a reduction in gait velocity is a concern. This information can then be utilized to design targeted interventions aimed at improving balance in the elderly population, where decreased gait velocity is strongly associated with an increased risk of falls and reduced mobility. Such interventions may involve prescribing specific cycling protocols or exercises, and cycling interventions could be calibrated to ensure the participant achieves at least a 30% increase in cadence, thereby meeting the threshold for measurable improvements in walking performance. In turn, this could effectively enhance gait velocity and, consequently, enhance balance and reduce the risk of falls in older individuals.

Several limitations should be acknowledged when interpreting the results of our study. Firstly, our research focused solely on a specific age group, specifically younger adults aged 18–39 years. Consequently, the generalizability of our findings to the older adult population may be restricted. Secondly, the use of straps to secure reflective markers on participants’ bodies posed an additional limitation. This approach may have introduced soft-tissue artifacts, potentially influenced the trajectories of the markers, and subsequently affected the accuracy of our measurements. Additionally, because our participants were randomly assigned to one of three groups, our results only support the effects of increased gait velocity after a single bout of cycling at a single cadence increase. Lastly, it is important to recognize that our study was conducted within controlled laboratory settings. Our results demonstrate an acute and immediate increase in gait velocity, and do not measure any lasting or rehabilitating effects. Therefore, the extent to which our results can be generalized to the community-dwelling environment may be limited.

## 5. Conclusions

Our findings indicate that a minimum increment in cycling cadence to above 30% of gait cadence is necessary to achieve a meaningful acute improvement in gait velocity. These results have potential implications for improving gait velocity in populations where reductions in gait velocity matter. Implementing interventions that focus on increasing cycling cadence could prove to be beneficial in enhancing gait velocity among older individuals if future studies confirm these results in this population. Future research could explore the long-term dose–response effects of this intervention and its application across different rehabilitation protocols. Additionally, a long-term objective would be to assess whether these acute adaptations can be maintained chronically to determine their practical utility for rehabilitation and balance.

## Figures and Tables

**Table 1:** Participant demographic information in the TEN, TWENTY, and THIRTY groups, and gait velocities for all groups, presented as mean ± s.d.

	TEN (*n* = 14)	TWENTY (*n* = 14)	THIRTY (*n* = 14)	*p* (η^2^)
Age (years)	21.86 ± 5.25	22.57 ± 3.99	23.29 ± 3.85	0.695 (0.125)
Mass (kg)	62.35 ± 13.99	73.77 ± 16.42	76.53 ± 14.37	0.059 (0.330)
Height (m)	1.69 ± 0.08	1.72 ± 0.09	1.75 ± 0.13	0.350 (0.197)
BMI (kg/m^2^)	21.77 ± 4.54	24.9 ± 4.6	24.91 ± 2.76	0.074 (0.298)

*p* = *p*-value, η^2^ = eta squared.

**Table 2:** PRE-to-POST change in spatiotemporal metrics of TEN, TWENTY, and THIRTY groups post-cycling (presented as mean ± s.d).

	TEN	TWENTY	THIRTY	*p* (η^2^)
Double Limb Support Time (s)	−0.01 ± 0.01	−0.00 ± 0.01	−0.03 ± 0.04 *	**0.022** (0.356)
Stride Width (m)	0.00 ± 0.18	0.00 ± 0.03	0.00 ± 0.02	0.734 (0.116)
Stride Length (m)	0.02 ± 0.03	0.02 ± 0.03	0.04 ± 0.12	0.733 (0.117)
Overall Cadence (SPM)	2.74 ± 2.6	2.54 ± 3.36	5.42 ± 4.83 *^,#^	**0.035** (**0.290**)
Gait Velocity (m/s)	0.03 ± 0.03	0.04 ± 0.05	0.07 ± 0.04 *^,#^	**0.031** (**0.604**)

* = significantly different from TEN; ^#^ = significantly different from TWENTY; *p* = *p*-value; η^2^ = eta squared; SPM = steps per minute; **Bold** indicates statistical significance.

**Table 3:** Absolute change in gait velocity (m/s) of males (m) and females (f) in each group, presented as mean ± s.d.

	TEN m = 7 f = 7	TWENTY m = 8 f = 6	THIRTY m = 6 f = 8
Male	0.02 ± 0.03	0.04 ± 0.05	0.06 ± 0.04
Female	0.04 ± 0.03	0.04 ± 0.04	0.09 ± 0.04
*p*	0.278	0.171	0.400

*p* = *p*-value.

**Table 4:** PRE-to-POST change in ground reaction force (GRF) during the stance phase of gait presented as mean ± s.d. normalized to body weight (BW).

	TEN	TWENTY	THIRTY	*p* (η^2^)
Peak Braking GRF	−0.001 ± 0.25	0.023 ± 0.04	−0.001 ± 0.03	0.079 (0.294)
Peak Propulsive GRF	0.006 ± 0.03	0.012 ± 0.02	0.013 ± 0.01	0.645 (0.136)
Peak Vertical LR GRF	0.017 ± 0.04 ^#^	−0.028 ± 0.06	0.018 ± 0.04 ^#^	**0.004 (0.332)**
Peak Vertical PO GRF	0.037 ± 0.04	0.031 ± 0.04	0.022 ± 0.04	0.632 (0.176)

^#^ = significantly different from TWENTY; *p* = *p*-value, η^2^ = eta squared, LR = Loading response or first peak vertical GRF. PO = push-off, or second peak vertical GRF. **Bold** indicates statistical significance.

**Table 5:** PRE-to-POST change in peak sagittal plane stance-phase joint angles during one stride of the right leg presented as mean ± s.d., reported in degrees.

	TEN	TWENTY	THIRTY	*p* (η^2^)
Peak Plantarflexion	−0.27 ± 2.6	−2.09 ± 2.8	1.32 ± 4.9	0.053 (0.315)
Peak Dorsiflexion	−0.48 ± 2.5	−1.43 ± 4.7	1.08 ± 2.2	0.148 (0.258)
Peak Knee Flexion	0.00 ± 3.8	−0.40 ± 2.4	−0.05 ± 2.4	0.966 (0.015)
Peak Knee Extension	−1.04 ± 3.6	0.65 ± 7.1	−0.46 ± 3.2	0.662 (0.132)
Peak Hip Flexion	−1.07 ± 6.6	1.89 ± 6.1	0.77 ± 3.7	0.412 (0.184)
Peak Hip Extension	−0.97 ± 6.5	1.95 ± 5.1	0.70 ± 5.6	0.382 (0.190)

*p* = *p*-value, η^2^ = eta squared.

**Table 6:** PRE-to-POST change in peak sagittal plane stance-phase joint moments during the stance phase of gait presented as mean ± s.d. reported in Nm/kg.

	TEN	TWENTY	THIRTY	*p* (η^2^)
Peak Plantarflexion	−0.03 ± 0.08	−0.07 ± 0.07	−0.03 ± 0.05	0.317 (0.206)
Peak Dorsiflexion	0.01 ± 0.06	−0.04 ± 0.06	−0.01 ± 0.05	0.054 (0.314)
Peak Knee Flexion	−0.02 ± 0.11	−0.03 ± 0.10	−0.03 ± 0.05	0.966 (0.014)
Peak Knee Extension	0.02 ± 0.08 ^#^	−0.12 ± 0.17	−0.01 ± 0.11 ^#^	**0.009 (0.393)**
Peak Hip Flexion	0.05 ± 0.14	0.06 ± 0.22	0.06 ± 0.16	0.206 (0.031)
Peak Hip Extension	−0.15 ± 0.21	−0.5 ± 0.17	−0.08 ± 0.18	0.952 (0.237)

^#^ = significantly different from TWENTY; *p* = *p*-value η^2^ = eta squared. **Bold** indicates statistical significance.

## Data Availability

Dataset available on request from the authors.
